# Erythropoietin suppresses the activation of pro-apoptotic genes in head and neck squamous cell carcinoma xenografts exposed to surgical trauma

**DOI:** 10.1186/1471-2407-14-648

**Published:** 2014-09-02

**Authors:** Gustaf Lindgren, Lars Ekblad, Johan Vallon-Christersson, Elisabeth Kjellén, Maria Gebre-Medhin, Johan Wennerberg

**Affiliations:** Department of Otorhinolaryngology/Head and Neck Surgery, Lund University Hospital, SE-22185 Lund, Sweden; Department of Oncology, Lund University Hospital, Lund, Sweden

**Keywords:** Erythropoietin, Head and neck cancer, Surgery, Apoptosis, Wound healing, Xenograft

## Abstract

**Background:**

Several studies on the use of erythropoietin (Epo) to treat anaemia in patients undergoing cancer treatment have shown adverse effects on tumour control and survival. Experimental studies indicate that this could be linked to an interaction with wound healing processes and not an effect on tumour cells *per se*. We have previously shown that erythropoietin in combination with surgical trauma stimulates tumour growth. In the present study, we investigated the effect of surgery and Epo on gene expression.

**Methods:**

Human tumours from oral squamous cell cancer were xenotransplanted to nude mice treated with Epo. The tumours were then transected in a standardised procedure to mimic surgical trauma and the change in gene expression of the tumours was investigated by microarray analysis. qRT-PCR was used to measure the levels of mRNAs of pro-apoptotic genes. The frequency of apoptosis in the tumours was assessed using immunohistochemistry for caspase-3.

**Results:**

There was little change in the expression of genes involved in tumour growth and angiogenesis but a significant down-regulation of the expression of genes involved in apoptosis. This effect on apoptosis was confirmed by a general decrease in the expression of mRNA for selected pro-apoptotic genes. Epo-treated tumours had a significantly lower frequency of apoptosis as measured by immunohistochemistry for caspase 3.

**Conclusions:**

Our results suggest that the increased tumour growth during erythropoietin treatment might be due to inhibition of apoptosis, an effect that becomes significant during tissue damage such as surgery.

This further suggests that the decreased survival during erythropoietin treatment might be due to inhibition of apoptosis.

**Electronic supplementary material:**

The online version of this article (doi:10.1186/1471-2407-14-648) contains supplementary material, which is available to authorized users.

## Background

Squamous cell carcinoma of the head and neck (HNSCC) is globally a common disease. Annually, more than 147,500 cases and 63,300 attributed deaths are reported in Europe [1, 2] and the prognosis for clinically advanced cancer is still very poor. It often affects patients with severe co-morbidity and both the cancer and the treatment, such as surgery, radiotherapy, chemotherapy and combinations thereof, have strong adverse effects on the patient’s general condition and nutritional status. Weight loss and anaemia are common. It has been argued that increased blood flow and oxygenation in the tumours would make them more accessible to radiotherapy and chemotherapy [3–5]. Erythropoietin (Epo) has been advocated to increase haemoglobin concentrations with the intent of improving the effect of radiotherapy and the quality of life.

Early studies of Epo treatment in cancer patients primarily investigated the effects on haemoglobin level [3–6] and quality of life [7]. Few studies had tumour growth, disease free survival and overall survival as primary endpoints. In 2003, a study [8] revealed significantly worse outcome for HNSCC patients treated with Epo. Other studies involving Epo administration during treatment of non-small-cell carcinoma of the lung (NSCLC) [7] and breast cancer [9] also showed lower survival rates for Epo treated patients. These results raised the concern that Epo might stimulate tumour growth. Epo has also been implicated in tumour invasiveness [10–12]. Several studies on the use of Epo to ameliorate anaemia in patients undergoing cancer treatment have shown adverse effects on tumour control and survival.

We have previously shown that Epo in combination with surgical trauma can stimulate growth of xenotransplanted tumours [13], while there was no growth stimulating effect of Epo alone. Later, we showed that the combination effect of Epo and surgery did not involve a direct interaction between Epo and the tumour cells [14].

In the present work, we analysed xenografted tumours using DNA microarrays in order to establish which cellular pathways that might be affected by Epo when combined with surgery.

## Methods

### Tumour line

The tumour line LU-HNSCC-7 was originally established from a moderately differentiated squamous cell carcinoma of the bucca (T2N0M0). It is aneuploid and without p53 mutation or cyclin D1 gene amplification [15].

### Establishment of xenograft

The study was approved by the Swedish National Board for Care of Laboratory Animals (M-48-06). The xenografts were established using a previously described method [16]. Tumour sample from the tumour line LU-HNSCC-7 were inoculated subcutaneously in the flank of BALB/c nude mice. Tumour volume was calculated from orthogonal diameter measurements every two or three days using the formula:


Where *V* = volume. *L* = length, and *W* = width

The mice were also weighed regularly. Tumours with a volume of smaller than 40 mm^3^ or greater than 300 mm^3^ at the time of surgery were excluded from the analysis, so were animals showing weight loss in order to ensure undisturbed logarithmic growth.

### Administration of erythropoietin

Recombinant human Epo (NeoRecormone, Roche; 400 IU/kg body weight) or physiological saline (placebo) was administered by subcutaneous injection (10 μL/g body weight) every third day starting from the day of transplantation.

### Surgical procedure and sampling of tumours

**Figure 1:**
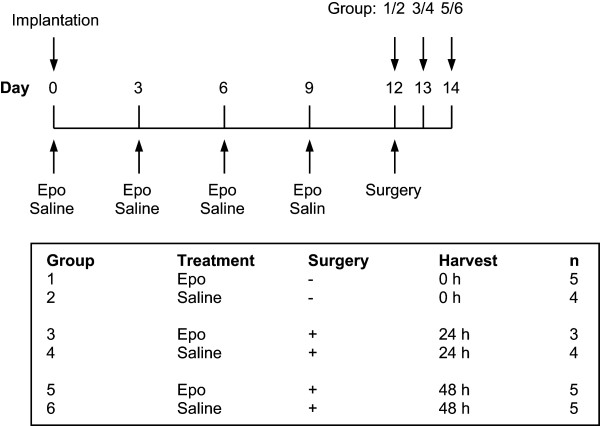
**Microarray analysis of six groups: group 1–2 no surgery +/-Epo; group 3–6 +/-surgery after 24 and 48 hours respectively.** There were five tumours per group but a total of four tumours were excluded.

Tumour bearing mice were treated with subcutaneous injections of Epo (NeoRecormone, Roche; 400 IU/kg body weight) or physiological saline (placebo) (10 μL/g body weight) every third day starting from the day of transplantation (Figure 1). After 12 days, the tumours were subjected to a standardised surgical trauma with a subcutaneous transection of the tumour using an injection needle. The tumours were collected for analysis at the indicated time points after surgery. Separate sets of tumours were established in an identical manner for the analysis of mRNA by microarray and qRT-PCR, and for the analysis of apoptosis.

### Histological verification

The establishment of the solid malignant xenografts was confirmed using histological examination with hematoxylin and eosin staining performed in conjunction with the harvesting of tumours.

### Microarrays

RNA was extracted from the tumour samples and microarray hybridisation was performed using the Illumina Human-6 Expression BeadChip KitVersion-2 (Illumina Inc., San Diego, CA, USA). The scanning was performed on Illumina Bead Array Reader (Illumina Inc., San Diego, CA, USA). The analysis of the fluorescent signals was performed using Multiexperiment Viewer software (MeV, Dana-Farber Cancer Institute, Boston, MA).

### Quantification of mRNA by qRT-PCR

Extraction of RNA was done with the AllPrep DNA/RNA Mini kit from Qiagen (Hilden, Germany) according to the manufacturer’s instructions. The expression of mRNA was measured by TaqMan gene expression assays from Applied Biosystems (Carlsbad, CA, USA) (DIP, BCL2L13, CASP1, MIF, CARD10, AIFM1, BIK, and BID with FAM labelled probes, ID: Hs00209789_m1, Hs00354836_m1, Hs00354836_m1, Hs00236988_g1, Hs00367225_m1, Hs00377585_m1, Hs00609635_m1, and Hs00609632_m1 respectively, and GAPDH with a VIC labelled probe, cat. no: 4326317E) with the Rotor-Gene Multiplex RT-PCR kit (QIAGEN, Hilden, Germany) in a Rotor-Gene RG-3000 (Corbette Research, St. Neots, UK) with the following program: reverse transcription 15 min, 50°C followed by 5 min at 95°C and then 15 s at 95°C and 15 s at 60°C in 40 cycles.

### Immunohistochemical analysis of apoptosis

Tumours were cut in 4-μm sections and stained using the TechMate 500 autostainer (Ventana Biotek, Tucson, AZ, USA). The primary antibody was anti-active caspase-3 antibody (cat. no. AF835, R&D Systems, Minneapolis, MN, USA). ChemMate EnVision Detection Kit (DakoCytomation, Glostrup, Denmark) was used for detection. In each of the tumour samples, the number of stained apoptotic cells was counted in three fields with a 40× objective.

### Statistical methods

**Figure 2:**
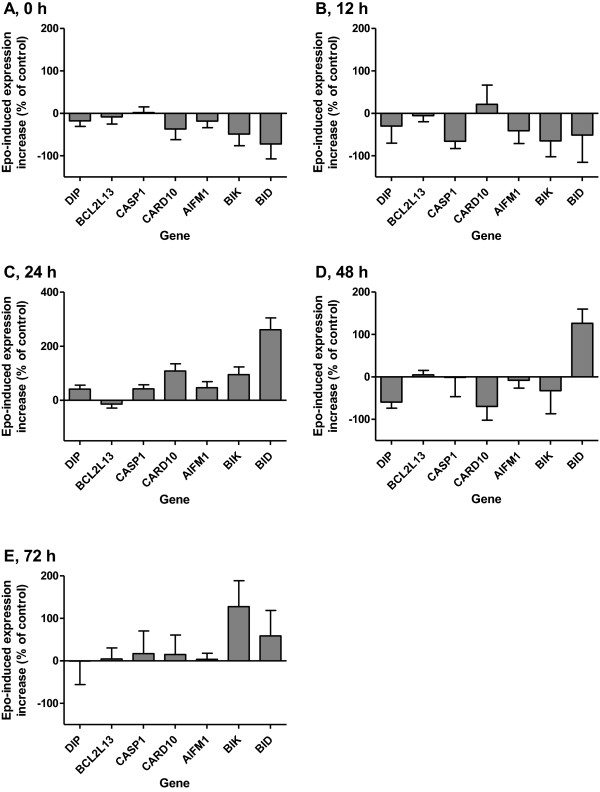
**qRT-PCR analysis of pro-apoptotic genes.** The bars show the increase in gene expression in Epo- compared to placebo-treated tumours measured after **A**. 1 h (P < 0.0001), **B**. 12 h (P = 0.0005), **C**. 24 h (P = 0.0003), **D**. 48 h (P = 0.66), and **E**. 72 h (P = 0.20). The influence of Epo was analysed by 2-way ANOVA. Error bars represent SEM.

For the microarray analysis normalized data was filtered on a p-detection value <0.05. The intensities were log2 transformed and the rows were centred on the mean. A SAM 2-way ANOVA analysis for Epo-significant genes was performed for the surgery groups using the results of the untreated tumours as reference (group, Figure 2). All tumours were compared using the DAVID (Database for Annotation, Visualization and Integrated Discovery) [17, 18] functional clustering tool. The likelihood of an enrichment of the genes involved in different biological pathways and themes was determined using EASE-score [19] which is a modified and more stringent form of Fisher Exact P-Value.

The qRT-PCR results were analysed by 2-way ANOVA and in the apoptosis analysis the groups were compared using an independent samples Mann–Whitney test.

## Results

### Microarray analysis

For the DNA microarray analysis, five tumours were set up in a total of six groups – three receiving Epo and three placebo (Figure 1). Of the tumours, three were excluded as the tumour sizes were outside the set limits and one due to failed hybridization. The tumours were analysed at two time points, 24 and 48 hours after surgery, since our previous results showed that the main effect of Epo ended within 48 hours after surgery and we were interested in early processes within this interval [13]. A total of 13,461 genes were analyzed. The microarray data was deposited at the Gene Expression Omnibus (GEO) at the National Center for Biotechnology Information (NCBI) (see Additional file 1).

In line with previous results, showing that the cell line LU-HNSCC-7 does not express the Epo receptor (EpoR) [14], this receptor was not significantly expressed in the microarray samples. This was also true for Epo.

A combined 2-way ANOVA analysis of all groups having undergone surgery (group 3–6) showed 1371 Epo-significant genes.

The functional clustering tool DAVID was used to analyse the enrichment of differentially expressed genes in cellular pathways when comparing Epo versus non-Epo treated tumours at the different time points after surgery. Many pathways were significantly enriched at one or several of the time points. However, we focussed the analysis on pathways that might be of importance for tumour growth. None of these pathways were enriched when comparing the control tumours that had not been subjected to surgery (data not shown). For the surgery groups tumour growth, cell cycle control and angiogenesis were not enriched (Table 1). However, there was a significant enrichment of genes involved in apoptotic pathways, both 24 and 48 hours after surgery (Table 1). A further analysis of the differentially expressed genes included in these pathways showed that pro-apoptotic genes tended to be down-regulated.

**Table 1:** **Analysis of the gene expression data using the DAVID functional annotation tool**

	24 h		48 h		24 and 48 h	
Biological theme (pathway)	Genes (No)	P-value	Genes (No)	P-value	Genes (No)	P-value
Apoptosis	81	6.0 × 10^-4^	97	3.0 × 10^-2^	20	1.5 × 10^-4^
Programmed cell death	82	6.0 × 10^-4^	99	6.0 × 10^-4^	19	3.7 × 10^-4^
VEGF-signalling	8	5.5 × 10^-1^	12	2.5 × 10^-1^	0	n.a.
Angiogenesis	9	9.7 × 10^-1^	0	n.a.	0	n.a.
Blood vessel development	20	8.3 × 10^-1^	0	n.a.	0	n.a.
Response to hypoxia	0	n.a.	0	n.a.	0	n.a.

NOTE The table shows the number of genes with significantly altered expression involved in pathways related to angiogenesis, hypoxia and apoptosis. The p-value was defined as the EASE score which is a more stringent form of Fishers exact p-value.

### Quantification of mRNA by qRT-PCR

To verify the down-regulation of pro-apoptotic genes seen in the microarray analysis we selected 7 genes involved in apoptosis (DIP, BCL2L13, CASP1, CARD10, AIFM1, BIK and BID), all displaying decreased expression in the DNA microarray analysis, and performed qRT-PCR to measure the gene expression. This analysis was performed on a separate set of tumours that had not been included in the microarray analysis. Twelve groups of tumours were analysed: two groups each (+/-Epo) 1, 12, 24, 48, and 72 hours after surgical transection.

We chose to collect tumours 12 hours after surgery presuming that the effect on transcription would precede the response at protein and cellular levels. There was no effect after 72 hours (data not shown).

We analysed the mRNA level for the following genes, which all displayed decreased expression in the DNA microarray analysis: DIP, BCL2L13, CASP1, CARD10, AIFM1, BIK and BID. The expression of the set of pro-apoptotic genes was significantly decreased at the 1 and 12 hour time points (P < 0.0001 and P = 0.0005 respectively). At 24 hours there was an increase in expression (P = 0.0003), while there was no significant difference at 48 and 72 hours (Figure 2). This indicates an early decrease in the expression of pro-apoptotic genes, followed by a transient up-regulation 24 hours after surgery and then returning to basal expression after 48 hours.

### Immunohistochemical assessment of apoptosis

**Figure 3:**
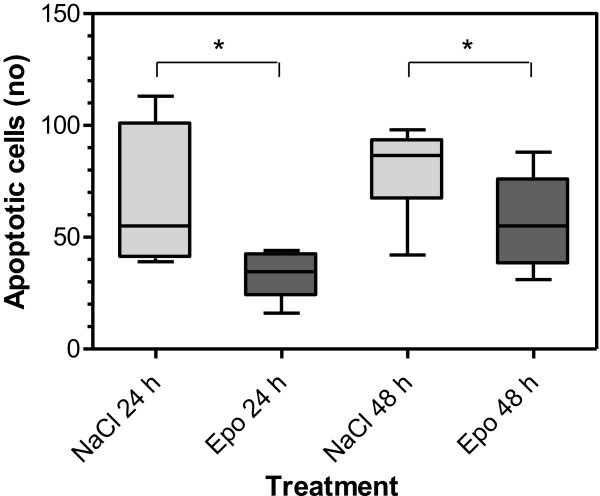
**Analysis of apoptosis using immunohistochemistry for caspase-3.** The combined number of apoptoses counted per tumour sample in Epo- and placebo-treated groups 24 and 48 hours after surgery respectively.

**Figure 4:**
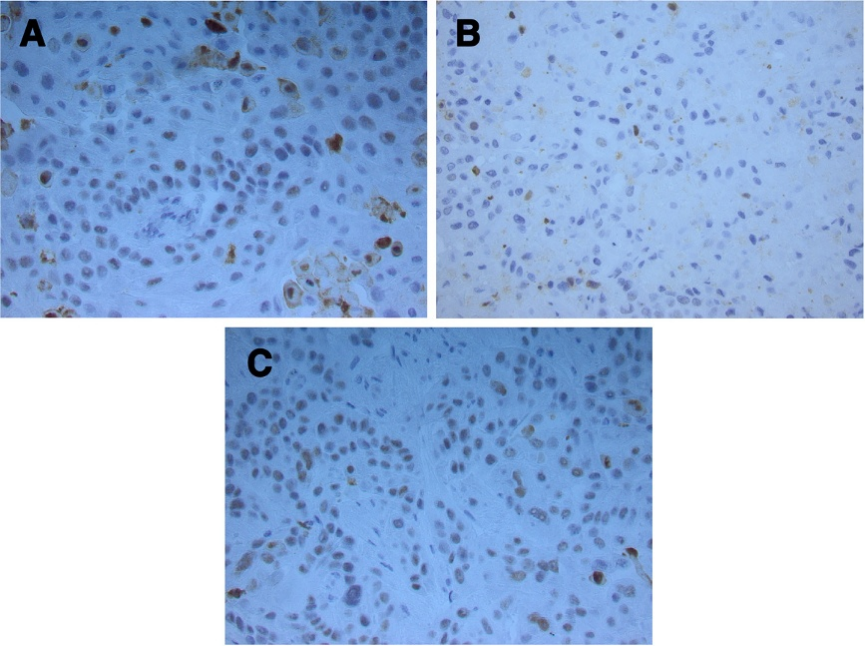
**Immunohistochemical staining for caspase 3 on surgically transected tumours. A)** Epo-treated tumour 24 hours after surgery. **B)** Placebo-treated tumour 24 hours after surgery. **C)** Placebo-treated tumour 48 hours after surgery

As a further confirmation of the effect on apoptosis, a separate set of tumours was analysed by immunohistochemistry. The expression of active caspase-3 was measured 24 and 48 h after surgery with or without Epo-treatment. There was a significant decrease in Caspase-3 expression in the Epo- compared with the placebo-treated tumours at both time points (P = 0.045 at 24 h and P = 0.030 at 48 h) (Figures 3 and 4).

## Discussion

Originally, the stimulating effect of erythropoietin on HNSCC has been assumed to involve angiogenesis and tumour hypoxia. Therefore, an initial assumption in this work was that pathways involved in these activities would be affected. However, we found little change in the expression of genes involved in growth and angiogenesis but, on the other hand, we found an interesting decrease in the expression of pro-apoptotic genes. These results were further verified by qRT-PCR and immunohistochemical analysis of apoptosis.

In normal erythropoiesis, Epo has important apoptosis-inhibiting effects [20, 21] and it has also been shown to protect hypoxic neurons from apoptosis [22]. Earlier data suggest that this effect does not become apparent when Epo is given alone but when the tumour is subject to some kind of concurrent stress, for example cisplatinum treatment [5] or irradiation [23].

Surgical trauma can also stimulate proliferation through wound healing [24]. Many mechanisms in wound healing, e.g. paracrine growth factor signalling [25], angiogenesis [26] and DNA-replication initiation [27] are also disturbed in tumorigenesis, showing a close connection between the mechanisms of wound healing and tumour development and growth. We have previously seen increased tumour growth *in vivo* for this cell line after surgery while under Epo-treatment [13] but not at a cellular level *in vitro* [14], and the cell line was shown not to express the Epo receptor [14]. This suggests that Epo exerts its effect through interaction with stroma cells.

A hypothesis derived from the present study is that the early wound healing response (resulting from the surgical procedure) in combination with a secondary effect of Epo, mediated by stromal cells, suppresses the apoptotic potential within the tumour. The apoptosis-inhibiting effect of erythropoietin can be the common mechanism for the increased tumour survival when it is combined with any treatment – surgery, radiation or chemotherapy.

Wound healing is a long multi-stage process involving inflammatory, proliferative and proliferative phases. In this study, we focused on the early inflammatory phase since it was during the first 48 hours that we previously had seen a growth delay in this model [13].

Our findings have important clinical consequences since this model of surgical trauma can be applicable to minimal residual disease after surgery. The remaining tumour tissue remains susceptible to the wound healing response in which Epo signalling plays a role [26]. It is particularly interesting considering the result of the study by Henke *et al*. since they found a particularly worse prognosis in their stratum 2, i.e. those patients who underwent incomplete surgery and received erythropoietin during postoperative radiotherapy [8]. It must be pointed out that a diagnostic biopsy also induces a surgical trauma and a subsequent wound healing process. The result also underlines the possibility of an anti-apoptotic approach in future cancer treatment.

## Conclusions

For the understanding of tumour survival and growth, we must not only consider the innate properties of the tumour cells. We must also take into account the almost parasitic approach with which the tumour interacts with the surrounding stroma.

Surgery damages tissue and triggers a stressful wound healing response. The use of antiapoptotic substances, such as Epo, increases tumour cell survival when the tissue is under stress. The use of Epo to patients undergoing tumour treatment, including surgery, is therefore counterproductive and possibly hazardous.

## Electronic supplementary material

Additional file 1: **Microarray data are deposited and available at the Gene Expression Omnibus (GEO) at the National Center for Biotechnology Information (NCBI).** http://www.ncbi.nlm.nih.gov/geo/query/acc.cgi?acc=GSE58194. (ZIP 22 MB)
